# Comparative Analysis of *Panax ginseng* Berries from Seven Cultivars Using UPLC-QTOF/MS and NMR-Based Metabolic Profiling

**DOI:** 10.3390/biom9090424

**Published:** 2019-08-28

**Authors:** Dahye Yoon, Bo-Ram Choi, Young-Chang Kim, Seon Min Oh, Hyoung-Geun Kim, Jang-Uk Kim, Nam-In Baek, Suhkmann Kim, Dae Young Lee

**Affiliations:** 1 Department of Herbal Crop Research, National Institute of Horticultural and Herbal Science, RDA, Eumseong 27709, Korea; 2 Department of Oriental Medicine Biotechnology, Kyung Hee University, Yongin 17104, Korea; 3 Department of Chemistry, Center for Proteome Biophysics, and Chemistry Institute for Functional Materials, Pusan National University, Busan 46241, Korea

**Keywords:** Ginseng berries, UPLC-QTOF/MS, HR-MAS NMR spectroscopy, ginsenoside, primary metabolite

## Abstract

The commercial use of *Panax ginseng* berries is increasing as *P. ginseng* berries are known to contain large amounts of ginsenosides, and many pharmacological activities have been reported for the various ginsenosides. For the proper use of *P. ginseng* berries, it is necessary to study efficient and accurate quality control and the profiling of the overall composition of each cultivar. Ginseng berry samples from seven cultivars (Eumseung, Chung-buk Province, Republic of Korea) were analyzed using ultra-performance liquid chromatography-quadrupole-time-of-flight mass spectrometry (UPLC-QTOF/MS) for profiling of the ginsenosides, and high-resolution magic-angle-spinning nuclear magnetic resonance (HR-MAS NMR) spectroscopy for profiling of the primary metabolites. Comparing twenty-six ginsenoside profiles between the variant representatives and between the violet-stem variant, Kumpoong and Sunwon were classified. In the case of primary metabolites, the cultivars Kumpoong and Gopoong were classified. As a result of correlation analyses of the primary and secondary metabolites, in the Gopoong cultivar, the metabolism was found to lean toward energy metabolism rather than ginsenoside synthesis, and accumulation of osmolytes was low. The Gopoong cultivar had higher levels of most of the amino acids, such as arginine, phenylalanine, isoleucine, threonine, and valine, and it contained the highest level of choline and the lowest level of myo-inositol. Except for these, there were no significant differences of primary metabolites. In the Kumpoong cultivar, the protopanaxatriol (PPT)-type ginsenosides, ginsenoside Re and ginsenoside Rg2, were much lower than in the other cultivars, while the other PPT-type ginsenosides were inversely found in much higher amounts than in other cultivars. The Sunwon cultivar showed that variations of PPT-type ginsenosides were significantly different between samples. However, the median values of PPT-type ginsenosides of Sunwon showed similar levels to those of Kumpoong. The difference in primary metabolites used for metabolism for survival was found to be small in our results. Our data demonstrated the characteristics of each cultivar using profiling data of the primary and secondary metabolites, especially for Gopoong, Kumpoong, and Sunwon. These profiling data provided important information for further research and commercial use.

## 1. Introduction

*Panax ginseng* C.A. Mey. is one of the most important herbal products today, and its root has been widely used as a constituent of traditional medicine in Korea and other countries [1].

*P. ginseng* root contains diverse bioactive compounds, and ginsenosides are the major components. Previously, a number of studies have reported that ginsenosides show various pharmacological properties such as antitumor, antidiabetes, antifatigue, antistress, antioxidative, and antiaging effects, as well as enhancement of the immune system [2,3,4,5]. 

Due to their utility, the interest in the therapeutic potential of ginsenosides has increased. Ginsenosides are distributed in several parts of the *P. ginseng* plant, including not only the root but also the leaves and berries. Different parts of the plant contain distinct constituent compositions, and therefore may have different pharmacological activities [6,7,8]. 

Most of the previous reports on this topic have focused on the ginseng root as a reservoir of ginsenosides. Recent studies have also demonstrated that ginseng berries have a high content of ginsenosides, and their own various pharmacological properties [9,10,11,12].

Dey et al. compared the antihyperglycemic effect of both ginseng root and ginseng berries. They found that the berry exhibits more potent antihyperglycemic activity than the root, and only the berry shows marked antiobesity effects in mice [13].

Moreover, ginseng berry extract and compounds show potent antioxidative and antitumor activities [12,14,15].

In order to provide new insights for future study into *P. ginseng* berries, Lee et al. reviewed the chemical constituents and biological activities of the ginseng berry [16].

For the phytochemical study and quality control of ginseng berries, it is critical to assess the ginsenoside content in the berries. Analytical methods for assessing ginsenosides have been established by high-performance liquid chromatography (HPLC) coupled with a UV detector [6,17], or an evaporative light-scattering detector (ELSD) [18]. 

However, this method is limited to the simultaneous analysis of multiple target compounds. Additionally, such methods still lack the required sensitivity and selectivity to profile various ginsenosides. Recently, liquid chromatography (LC) coupled with mass spectrometry (MS) has emerged as a good tool for the sensitive and selective analysis of various ginsenosides. In particular, a quadrupole-time-of-flight (QTOF)/MS with ultra-performance liquid chromatography (UPLC) system is useful for the rapid and high-resolution separation of ginsenosides in ginseng [8,19,20].

The secondary metabolites of many plants have been studied extensively because they are characterized by the influence of plant characteristics and the environment. Although the primary metabolites are essential for growth and development, there is relatively less research focused on them compared to secondary metabolites. However, these secondary metabolites are produced from the primary metabolites. The study of primary metabolites, as well as secondary metabolites, is important in phytochemical research. For screening the major primary metabolites of a powdered sample, high-resolution magic-angle-spinning nuclear magnetic resonance (HR-MAS NMR) spectroscopy is a good tool without requiring extraction of the sample. The dipolar, chemical shift anisotropy and magnetic susceptibility interactions vanish when a powdered sample is spun with a magic angle (54.7°). Therefore, a reduced line width, which is similar to the resolution of the solution, can be observed by HR-MAS NMR spectroscopy [21].

Thus, in this study, UPLC-QTOF/MS was used to perform a comprehensive profiling of ginsenosides from extract of the ginseng berry and HR-MAS NMR spectroscopy was used to identify and quantify the major primary metabolites from a powder of the ginseng berry. The ginsenoside content and the major primary metabolites of ginseng berries may differ depending on the cultivars. The comprehensive profiling of various ginsenosides and the primary metabolites of the ginseng varieties that are currently grown by the National Institute of Horticultural and Herbal Science (NIHHS), Chunpoong, Chungsun, Kumpoong, Yunpoong, Gopoong, Sunwon, and Sunun, have not yet been fully reported. The metabolite profiling and classification of seven cultivars with this comparative method is applicable to the evaluation and control of the quality of ginseng berries.

## 2. Materials and Methods 

### 2.1. Ginseng Berry Samples

**Figure 1:**
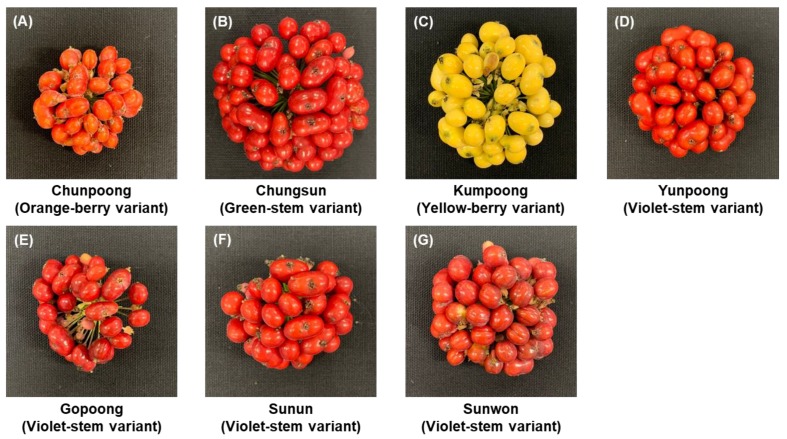
Characteristics of the fruit of varieties of ginseng. (**A**) Chunpoong. The color of the berries is orange. (**B**) Chungsun. The color of the berries is red. (**C**) Kumpoong. The color of the berries is yellow. (**D**) Yunpoong. The color of the berries is red. (**E**) Gopoong. The color of the berries is red. (**F**) Sunun. The color of the berries is red. (**G**) Sunwon. The color of the berries is red.

Six-year-old ginseng berries were cultivated in the field of the Department of Herbal Crop Research (DHCR) located in Eumseung, Chung-buk Province (127˚45ʹ013.14ʹʹE, 36˚56ʹ36.63ʹʹN), Republic of Korea. This cultivation protocol was followed by the “ginseng GAP standard cultivation guideline” developed by the Rural Development Administration (RDA), Republic of Korea. On July 25, 2017, ginseng berries, randomly selected from those available on this date, were collected from the 10–15 samples of six-year-old ginseng of the seven cultivars (Figure 1). The seeds were removed from the ginseng berries, and the fruits were crushed and stored at –80 °C and freeze-dried by freeze dryer under reduced pressure (–30 °C, 100 mTorr) for 24 h. A voucher specimen (NIHHS-201707) was deposited at the Herbarium of DHCR, NIHHS, RDA, Republic of Korea.

### 2.2. Standard Constituents and Reagents

HPLC-grade acetonitrile, methanol, and water were obtained from Merck (Darmstadt, Germany). Formic acid, deuterium oxide (D_2_O), and 3-(trimethylsilyl) propionic-2,2,3,3-*d_4_* acid sodium salt (TSP-*d_4_*) were purchased from Sigma-Aldrich (St. Louis, MO, USA). The ginsenosides were isolated and purified from *P. ginseng* roots by a series of chromatography procedures in our laboratory, and their structures were elucidated by comparison of spectroscopic data (MS, ^1^H-NMR, and ^13^C-NMR) with the literature data: ginsenoside Rb1, Ra2, Ra3, Rc, Ra1, Rb2, Rb3, Rd, F2, Rg3, Rh2, compound K, 20-O-glucoginsenoside Rf, ginsenoside Rg1, Rg2, Re, Rf, Rh1, notoginsenoside R2, ginsenoside F1, Rg6, Rk1, Ro, and Rk3, ginsenoside F4, Rh4, and Rg5 [8]. The purity of the isolated compounds was determined to be more than 98% by normalization of the peak areas detected by HPLC analysis.

### 2.3. Sample Preparation

Ginseng berries were lyophilized and ground (<0.5 mm) using a mixer (Hanil, Seoul, Korea) and thoroughly mixed, and subsamples were homogenized further using a Retsch MM400 mixer mill (Retsch GmbH, Haan, Germany) for later analyses.

For the UPLC-QTOF/MS analysis, fine powder was weighed (100 mg), suspended in 1 mL of 70% (*v/v*) methanol, and ultrasonically extracted for 1 h at 50 °C. After the centrifugation (13,500× *g*, 5 min at 4 °C), 900 µL of supernatant was transferred to a new Eppendorf tube. The solution was filtered through a syringe PTFE filter (0.2 µm) and diluted 20 times. Each sample was injected directly into the UPLC system.

For the HR-MAS NMR analysis, each powder sample was weighed to give 3 mg and transferred to the NMR nano tube (Agilent Technologies, Santa Clara, CA, USA). For the NMR analysis, 37 μL of D_2_O containing 2 mM TSP-*d_4_* was added to each NMR nanotube before the NMR measurement.

### 2.4. UPLC-QTOF/MS Analysis

UPLC was carried out on a Waters ACQUITY H-Class UPLC (Waters Corp, Milford, MA, USA) Chromatographic separations were performed on a Thermo Hypersil Gold column (2.1 mm × 100 mm, 1.9 µm). The column oven was maintained at 40 °C and the mobile phases consisted of solvent A [Water + 0.1% formic acid (*v/v*)] and solvent B [Acetonitrile + 0.1% formic acid (*v/v*)]. The elution gradient was as follows: 0–0.5 min, B 15%; 0.5–1 min, B 15–20%; 1–6 min, B 20%; 6–13 min, B 20–30%; 13–23 min, B 30–35%; 23–24 min, B 35–38%; 24–27 min, B 38–60%; 27–31 min, B 60–90%; 31–32 min, B 90–15%; 32–34 min, B 15%. The injection volume was 2 μL and the flow rate was 500 μL/min for each run. Methanol was run after every sample as a blank. Next, MS analysis was conducted on a Waters Xevo G2-S QTOF MS (Waters Corp.) in the negative ion mode. The mass spectrometers performed the MS^E^ acquisition mode with alternative high- and low-energy scans. The operational parameters were set as follows: source temperature, 120 °C; desolvation temperature, 550 °C; cone voltage, 40 V; capillary, 3.0 kV; cone gas flow, 30 L/h; and desolvation gas flow, 800 L/h. Accurate mass measurements were achieved with the use of an automatic calibration delivery system, which contained the internal reference [Leucine-enkephalin, *m/z* 556.276 (ESI+), *m/z* 554.262 (ESI-)]. Data were collected from *m/z* 100 to 2000 [8].

### 2.5. HR-MAS NMR Analysis

All spectra were acquired with a 600.167 MHz Agilent NMR spectrometer equipped with a 4 mm gHX NanoProbe (Agilent technologies, Santa Clara, CA, USA). The spinning rate was 2000 Hz. A total of 128 transients were collected using CPMG (Carr–Purcell–Meiboom–Gill) with a PRESAT pulse sequence for the suppression of water and high molecular mass compounds [22]. The spectra were obtained using 1.704 s of acquisition time, 1 s of relaxation delay, and 128 transients. The TSP-*d_4_* peak at 0.00 ppm was used for the reference to calibrate the chemical shift [23]. All spectra were phased and the baselines corrected manually. Metabolite assignment and quantification for each sample were processed using Chenomx NMR Suite 7.1 Professional (Chenomx Inc., Canada) and the Chenomx 600 MHz library database. For the multivariate statistical analysis, each NMR spectrum was binned from 0.5 to 8.0 ppm, and the water peak was excluded. The binning size was 0.001 ppm and normalization was conducted with total area. The binned spectra were aligned using the icoshift algorithm of MATLAB R2013b (The MathWorks, Natick, MA, USA). After the alignment, multivariate statistical analyses were conducted using SIMCA-P+ 12.0 software (Umetrics, Umeå, Sweden). The principal component analysis (PCA), partial least squares discriminant analysis (PLS-DA), and orthogonal partial least squares discriminant analysis (OPLS-DA) were processed.

## 3. Results

### 3.1. Construction of LC-MS Conditions to Profile Various Ginsenosides

For a comprehensive profiling of various ginsenosides from the *P. ginseng* berry, an analytical method based on UPLC-QTOF/MS was constructed. Extracts of natural products generally have complex metabolites, including known and unknown compounds. To identify the metabolites in a mixture, it is critical to measure the exact mass values of target metabolites. QTOF/MS, which provides a full mass scan at high resolution, is an effective way to analyze ginsenosides in a complex mixture. Among the various ginsenosides, there are several isomers that have the same mass values but different molecular structures. To determine the isomers, chromatographic separation is also required. A UPLC system with a small particle size column provides the rapid and effective separation of various compounds, including isomers. Thus, we used UPLC-QTOF/MS to profile various ginsenosides from the ginseng berry.

First, 37 standard samples of ginsenosides were used to optimize the LC-MS conditions. In the negative mode of electrospray ionization (ESI), the 37 ginsenosides were mainly detected as [M − H]^−^ and [M + COOH]^−^ ions. Under a flow rate of 500 µL/min using a Thermo Hypersil Gold column (2.1 mm × 100 mm, 1.9 µm particle size), various ginsenoside standards were separated well and eluted in 34 min. Although several compounds were eluted at almost the same times, the exact mass values could differentiate the ginsenosides. For a reliable and high-throughput analysis of various ginsenosides from many ginseng berry samples, it is required to construct an in-house library for the ginsenosides. The molecular formula and retention time (RT) of 37 ginsenosides were listed in the library. The *m/z* of ions from the ginsenoside profile data was automatically matched to the library compounds.

To construct the in-house library for the 37 ginsenosides, the molecular formula of each compound was added into the UNIFI software (v1.8, Waters Corp., Milford, MA, USA).

Next, we optimized the protocols to extract the ginsenosides from the ginseng berries. For effective extraction, it is critical to select a suitable solvent. Previously, several solvents such as water, ethanol (EtOH), and methanol (MeOH) have been widely used to extract ginsenosides from ginseng berries. First, we tried to compare the extraction efficiency of both EtOH and MeOH. A cultivar of ginseng berry, called Kumpoong, was used in this experiment. The EtOH or MeOH extracts of the ginseng berry were subjected to UPLC-QTOF/MS analysis. There was no significant difference between these two solvents, and we selected MeOH.

### 3.2. Profiling of Various Ginsenosides from Seven Different Cultivars of Ginseng Berries

**Figure 2:**
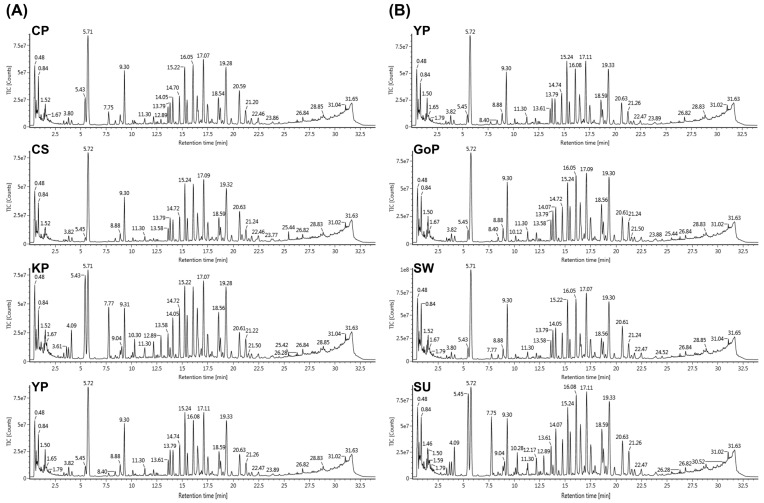
Representative ultra-performance liquid chromatography-quadrupole-time-of-flight mass spectrometry (UPLC–QTOF/MS) total ion chromatograms (TIC) of ginseng berry extracts. (**A**) Comparison of representative cultivars in each variant (CP, Chunpoong; CS, Chungsun; KP, Kumpoong; YP, Yunpoong). (**B**) Comparison of cultivars in the violet-stem variant (YP, Yunpoong; GoP, Gopoong; SW, Sunwon; SU, Sunun). See retention time in Table 1.

The UPLC-QTOF/MS-based metabolomics approach was applied to identify the ginsenosides. Total ion chromatograms were obtained from seven different cultivars of ginseng berries using optimized conditions for UPLC-QTOF/MS (Figure 2). 

**Figure 3:**
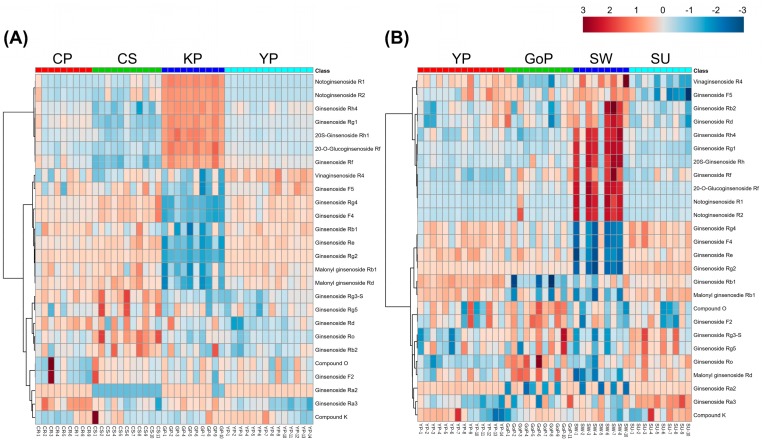
Heatmap of ginsenoside profiles from different cultivars. (**A**) Comparison of representative cultivars in each variant (CP, Chunpoong; CS, Chungsun; KP, Kumpoong; YP, Yunpoong). (**B**) Comparison of cultivars in the violet-stem variant (YP, Yunpoong; GoP, Gopoong; SW, Sunwon; SU, Sunun).

After metabolite profiling of individual samples by UPLC-QTOF/MS, each set of data was processed using the UNIFI software. Progenesis QI v2.3 (Nonlinear Dynamics, Newcastle, UK) was used for data processing, including peak picking, alignment, and univariate analysis for filtering the peaks as follows: fold change >2, and *p*-value < 0.05. In total, 5655 peaks were filtered, and then the peaks were matched with the ginsenoside library. Therefore, 26 ginsenosides were identified. Heatmap analysis was conducted with these ginsenoside profiles of representative cultivars in each variant (Figure 3A). Kumpoong in the yellow-berry variant showed a different pattern to the other cultivars, and Chunpoong in the orange-berry variant and Yunpoong in the violet-stem variant, which are native cultivars, had similar patterns to each other. On the other hand, in the results of heatmap analysis with four cultivars of the violet-stem variant, Sunwon showed patterns different from the other cultivars (Figure 3B).

### 3.3. Profiling of Primary Metabolites from Different Cultivars of Ginseng Berries Using HR-MAS NMR

**Figure 4:**
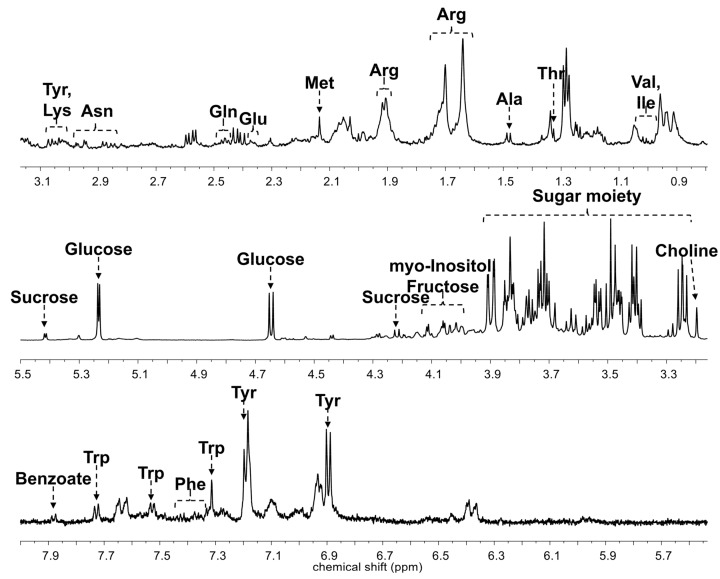
Representative ^1^H-nuclear magnetic resonance (NMR) spectrum with metabolite annotation. Ala, alanine; Arg, arginine; Asn, asparagine; Gln, glutamine; Glu, glutamate; Ile, isoleucine; Lys, lysine; Met, methionine; Phe, phenylalanine; Thr, threonine; Trp, tryptophan; Tyr, tyrosine; Val, valine.

The primary metabolites from seven different cultivars of ginseng berry were analyzed using HR-MAS NMR spectroscopy (Figures S1 and S2). The representative ^1^H-NMR spectrum is shown in Figure 4.

Twenty-four primary metabolites were identified and quantified, and a one-way analysis of variance (ANOVA) was conducted using MetaboAnalyst 4.0 (https://www.metaboanalyst.ca). In the results of the one-way ANOVAs on all cultivars, alanine, arginine, benzoate, choline, glucose, glutamate, lysine, methionine, phenylalanine, sucrose, tyrosine, valine, and myo-inositol were found to be the significant metabolites (*p*-value <0.05). In the comparison of the representative cultivars in each variant, glutamate, methionine, choline, glucose, asparagine, phenylalanine, and lysine were significant, and in the comparison of the four cultivars in the violet-stem variant, myo-inositol, alanine, sucrose, arginine, methionine, choline, valine, tyrosine, phenylalanine, glutamate, tryptophan, sn-glycero-3-phosphocholine, lysine, and fructose were significant.

**Figure 5:**
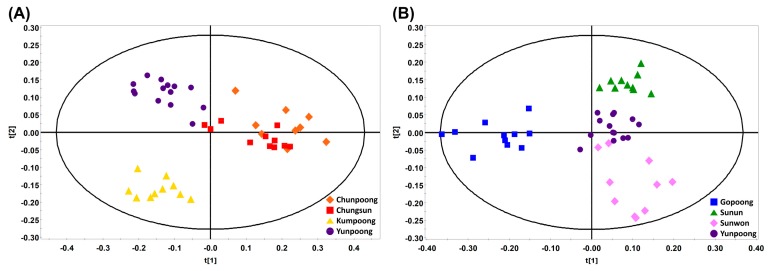
Multivariate statistical analyses of NMR spectra from different cultivars. (**A**) Comparison of representative cultivars in each variant (◆, Chunpoong; ■, Chungsun; ▲, Kumpoong; ●, Yunpoong). (**B**) Comparison of cultivars in violet-stem variant (■, Gopoong; ▲, Sunun; ◆, Sunwon; ●, Yunpoong).

Multivariate statistical analyses of the NMR spectra were conducted for a spectral pattern comparison of cultivars. Multivariate statistical analyses were performed on the Pareto-scaled NMR spectra of all samples. PCA, PLS-DA, and OPLS-DA were performed, but the clustering pattern was not good in the PCA models. PLS-DA and OPLS-DA showed the same clustering pattern, and OPLS-DA showed a clearer classification. Therefore, we presented the OPLS-DA score plots (Figure 5). The OPLS-DA score plot of representative cultivars in each variant showed the clustering of Chunpoong with Chungsun. Kumpoong was clearly differentiated. In the comparison of cultivars in the violet-stem variant, the score plot showed similar patterns for Sunun and Yunpoong. 

However, Gopoong showed a clear classification. These clustering patterns were different from the results of UPLC-QTOF/MS. From this, it was predicted that the perturbation patterns of primary and secondary metabolites differed according to the cultivars.

### 3.4. Pearson Correlation Analysis of Metabolites

**Figure 6:**
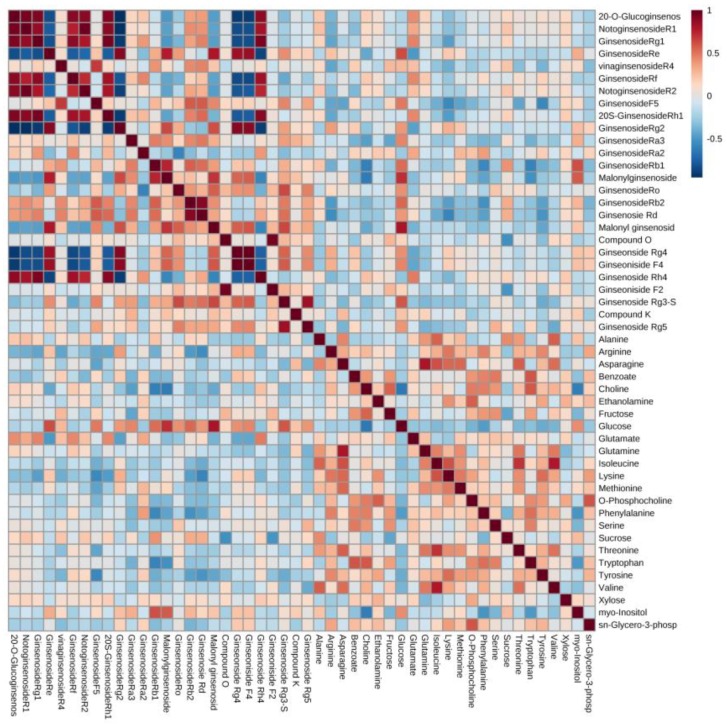
Correlation matrix of primary metabolites and ginsenosides. The scale bar shows the correlation coefficient from −1 to +1.

The results of clustering showed differences in the primary and secondary metabolites of different cultivars. These results suggest that the degree of metabolic pathway activation among cultivars may be different. Therefore, a Pearson correlation analysis was performed using MetaboAnalyst 4.0 (Xia Lab, McGill University, Montréal, Quebec, Canada) to confirm the correlation between the primary metabolites and the secondary metabolites, and to interrelate this with the metabolic pathway (Figure 6). Samples were normalized by a pooled sample from the Yunpoong cultivar, which is one of the native species. Log transformation was used to obtain a normal distribution. Data were scaled with Pareto scaling.

The range of the correlation coefficient (*r*) is from −1 to +1. In the case of a value less than 0, it is considered a negative correlation, and a value over 0 is considered a positive correlation [24]. The *r* value can be divided by the degree of correlation: 0.40–0.69, moderate correlation; 0.70–0.89, strong correlation; 0.90–1.00, very strong correlation [25].

## 4. Discussion

**Figure 7:**
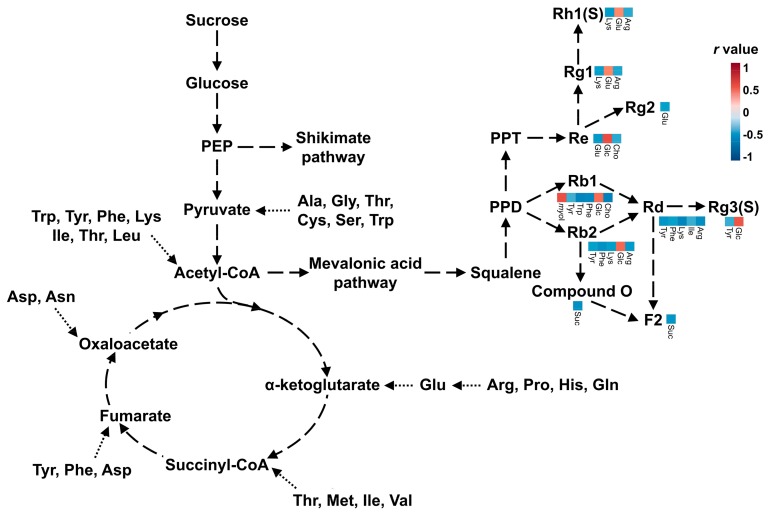
Pathway of energy metabolism and ginsenoside synthesis. Selected correlation matrix plots of primary metabolites with *r* > 0.4 are shown for each ginsenoside.

The purpose of this study was to investigate the metabolite profiles of various ginseng berry cultivars and to investigate the differences among the cultivars by profiling the primary metabolites, which are required for survival, and the ginsenosides, which are functional metabolites. From the analysis results, it was confirmed that the primary metabolites and ginsenosides were different among cultivars, and correlation analysis was performed to confirm the relationship between them. The *r* value obtained from the correlation analysis indicates the degree of correlation, and the interval is divided into specific standards. However, these standards are controversial, and researchers use different standards in various ways. In our results, the data was cut off based on the *r* value of 0.4. Most of the amino acids which had *r* values > 0.4 with ginsenosides showed negative correlations. Conversely, glucose had positive correlations with the ginsenosides, with an *r* value > 0.4. These metabolites are all on the energy metabolic pathway, and the cultivars that had abundant glucose had also abundant ginsenosides (Re, Rb1, Rb2, and Rg3(S)). The synthesis of ginsenosides requires acetyl-CoA which is formatted through glycolysis from glucose, therefore abundant glucose can make more ginsenosides. On the other hand, when acetyl-CoA is utilized in the synthesis of ginsenosides, amino acids should supplement the acetyl-CoA and TCA intermediates for normal energy metabolism. Therefore, ginsenosides have negative correlations with amino acids (Figure 7) [26].

**Figure 8:**
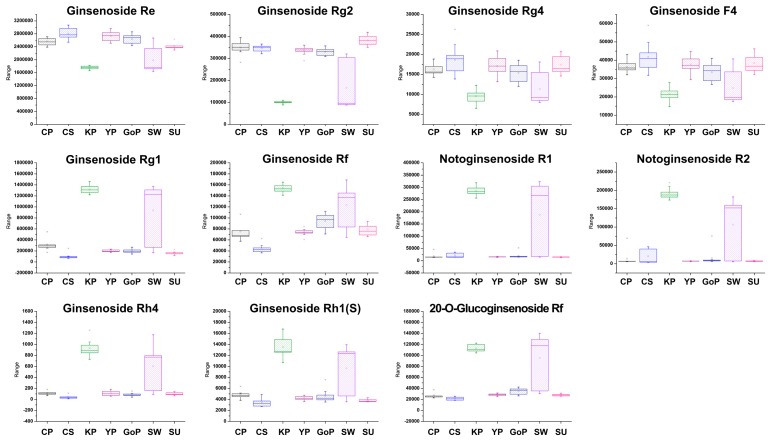
Box plots of protopanaxatriol (PPT)-type ginsenosides which showed characteristic patterns in the Kumpoong (KP) and Sunwon (SW) cultivars.

Sucrose synthesized from leaves is transported to nonphotosynthetic organs to supply energy and carbon skeletons [27]. Glucose is obtained from sucrose and used for synthesis of ginsenosides. Therefore, sucrose and ginsenosides showed negative correlations in our results. Choline is the precursor of glycinebetaine, which has the role of protecting plants from stress through the structural stabilization of complex proteins and membranes [28]. Myo-inositol is an osmolyte that plays an important role in phosphate storage, cell wall biosynthesis, stress-related molecular production, intercellular communication, and plant hormone transport [29]. From these results, choline and myo-inositol showed the opposite pattern of concentrations. Choline had a negative correlation with ginsenoside Rb1 and malonyl ginsenoside Rb1. On the other hand, myo-inositol had a positive correlation with them. These results were combined to link the more active metabolic pathway to the cultivars. The most specific cultivar was Gopoong. Most of the amino acids (arginine, phenylalanine, isoleucine, threonine, and valine) were present at higher levels in this cultivar, and most of the ginsenosides showed lower or similar levels than in the other cultivars. This seems to be due to normal energy metabolism rather than ginsenoside synthesis being prioritized in Gopoong. Gopoong contained the highest amount of choline among the cultivars, and the lowest amount of myo-inositol. It is thought to undergo less osmotic stress than the other cultivars. The other cultivars showed no significant differences in primary metabolites (Figure S3), and although the ginsenosides showed differences, they were not characteristic in the protopanaxadiol (PPD)-type (Figure S4). However, Kumpoong and Sunwon showed characteristic patterns in protopanaxatriol (PPT)-type ginsenosides (Figure 8). Notoginsenoside R1, R2, ginsenoside Rg1, Rh4, Rf, Rh1(S), and 20-*O*-glucoginsenoside Rf were present in higher levels in Kumpoong than in the other cultivars, while Re and Rg2 existed in much smaller amounts. In PPT-type ginsenosides, Sunwon showed a larger variation among samples than the other cultivars, however, the median values of these ginsenosides had levels similar to those of Kumpoong. Ginsenoside Rg4 and F4 were also weak, but showed patterns similar to Re and Rg2. Since there are few differences in the primary metabolites between cultivars, which are essential for general survival, there is little difference in the basic metabolism among the cultivars. However, the ginsenoside contents were different in each cultivar because of the different pathways and degree of response to the external environment in each cultivar.

These results can provide information on the cultivars that is useful for the production of pharmaceuticals, cosmetics, and so forth containing specific bioactivity. It is advantageous to use Chunpoong, Chungsun, Yunpoong, Gopoong, and Sunun for producing antidiabetic agents, based on the biological activity of ginsenoside Re [2,30,31,32]. On the other hand, the use of notoginsenoside R1, R2, ginsenoside Rg1, Rf, and Rh1-rich Kumpoong and Sunwon will be of benefit in the production of pharmaceuticals with neuroprotective effects [33,34,35,36,37,38,39,40] and cardioprotective effects [41,42,43,44,45].

## 5. Conclusions

This study was conducted to profile the overall metabolites of ginseng berries with pharmacological activities for commercial enhancement and quality control. There are various cultivars of ginseng berry, and representative cultivars of them were analyzed by UPLC-QTOF/MS and HR-MAS NMR spectroscopy for profiling of the primary and secondary metabolites. As a result, primary and secondary metabolites showed different patterns among cultivars. Therefore, the correlations between primary and secondary metabolites were analyzed. A characteristic metabolic pattern in Gopoong and the differential content of PPT-type ginsenosides in Kumpoong and Sunwon were confirmed. It was a new approach to the analysis of the correlation between primary and secondary metabolites in the ginseng berries. Through this study, it was possible to confirm the overall constituents by profiling the primary and secondary metabolites of the ginseng berry, and the characteristics of each variant were compared by confirming their differences. 

## Figures and Tables

**Table 1:** Relative percentages of ginsenosides in each cultivar (%).

No.	Expected RT (min)	Component Name	Formula	Observed *m/z*	Observed RT (min)	Adducts	Average of Relative Percentage (%)						
							CP	CS	KP	YP	GoP	SW	SU
1	3.6	20-O-Glucoginsenoside Rf	C48H82O19	1007.5265	3.62	+HCOO, -H	0.0732	0.0563	0.2943	0.0764	0.0899	0.2539	0.0787
2	4.07	Notoginsenoside R1	C47H80O18	977.5161	4.09	+HCOO, -H	0.0478	0.0570	0.7476	0.0409	0.0511	0.4965	0.0406
3	5.35	Ginsenoside Rg1	C42H72O14	845.4773	5.45	+HCOO, -H	0.8297	0.2613	3.4380	0.5507	0.5116	2.4831	0.4419
4	5.66	Ginsenoside Re	C48H82O18	991.5347	5.71	+HCOO, -H	6.7781	7.3395	4.5799	7.3372	6.7865	5.3904	6.7817
5	11.2	vinaginsenoside R4	C48H82O19	1007.5265	11.30	+HCOO, -H	0.2449	0.2195	0.2489	0.2805	0.2415	0.3290	0.2317
6	12.11	Ginsenoside Rf	C42H72O14	845.4770	12.17	+HCOO, -H	0.1992	0.1142	0.4025	0.1991	0.2414	0.3307	0.2202
7	12.81	Notoginsenoside R2	C41H70O13	815.4644	12.89	+HCOO, -H	0.0368	0.0528	0.5008	0.0193	0.0384	0.2823	0.0203
8	13.43	Ginsenoside F5	C41H70O13	815.4669	13.58	+HCOO, -H	0.4852	0.4637	0.4576	0.5050	0.4842	0.5796	0.4123
9	13.56	20(S)-Ginsenoside Rh1	C36H62O9	683.4246	13.66	+HCOO	0.0128	0.0090	0.0354	0.0114	0.0119	0.0258	0.0107
10	13.7	Ginsenoside Rg2	C42H72O13	829.4824	13.79	+HCOO, -H	0.9250	0.9064	0.2598	0.9042	0.8504	0.4588	1.0673
11	14.51	Ginsenoside Ra3	C59H100O27	1285.6185	14.27	+HCOO, -H	0.0033	0.0023	0.0032	0.0022	0.0026	0.0027	0.0031
12	14.35	Ginsenoside Ra2	C58H98O26	1255.6049	14.52	+HCOO, -H	0.0054	0	0.0057	0.0051	0.0031	0.0027	0.0038
13	14.53	Ginsenoside Rb1	C54H92O23	1153.5806	14.72	+HCOO, -H	0.5071	0.4411	0.4727	0.4999	0.3281	0.4326	0.4280
14	15.12	Malonyl ginsenoside Rb1	C57H94O26	1193.5794	15.24	-H	3.0830	2.9241	2.6016	3.1462	2.5714	2.5060	3.1080
15	15.69	Ginsenoside Ro	C48H76O19	955.4726	15.73	-H	0.0486	0.0705	0.0530	0.0437	0.0621	0.0506	0.0563
16	16.3	Ginsenoside Rb2	C53H90O22	1123.5710	16.48	+HCOO, -H	0.9968	1.0124	1.1320	0.9467	0.9372	1.1441	0.9034
17	18.34	Ginsenoside Rd	C48H82O18	991.5318	18.58	+HCOO, -H	1.6839	1.4716	1.7702	1.4030	1.4115	1.7513	1.3188
18	19.11	Malonyl ginsenoside Rd	C51H84O21	1031.5279	19.32	-H	5.0173	4.8832	4.3847	4.8414	5.1380	4.6440	4.9302
19	23.46	Ginsenoside Rg4	C42H70O12	811.4684	23.75	+HCOO, -H	0.0430	0.0489	0.0249	0.0459	0.0397	0.0310	0.0488
20	23.5	Compound O	C47H80O17	961.5136	23.77	+HCOO, -H	0.0930	0.0498	0.0527	0.0439	0.0610	0.0338	0.0317
21	24.28	Ginsenoside F4	C42H70O12	811.4695	24.52	+HCOO, -H	0.0964	0.1100	0.0566	0.1013	0.0864	0.0678	0.1077
22	24.68	Ginsenoside Rh4	C36H60O8	665.4140	24.90	+HCOO	0.0003	0.0001	0.0024	0.0003	0.0002	0.0016	0.0003
23	25.19	Ginsenoside F2	C42H72O13	829.4626	25.44	+HCOO, -H	0.0655	0.0346	0.0276	0.0238	0.0333	0.0201	0.0185
24	26.01	Ginsenoside Rg3-S	C42H72O13	829.4784	26.19	+HCOO, -H	0.0240	0.0252	0.0219	0.0198	0.0224	0.0199	0.0223
25	27.35	Compound K	C36H62O8	667.4320	27.54	+HCOO	0.0028	0.0060	0.0038	0.0039	0.0034	0.0038	0.0043
26	27.55	Ginsenoside Rg5	C42H70O12	811.4685	27.75	-H	0.0135	0.0153	0.0157	0.0135	0.0140	0.0134	0.0148

## Supplementary Materials

The following are available online at https://www.mdpi.com/2218-273X/9/9/424/s1, Figure S1: Representative ^1^H-NMR spectra of representative cultivars in each variant, Figure S2: Representative ^1^H-NMR spectra of cultivars in the violet-stem variant, Figure S3: Box plots of quantified primary metabolites, Figure S4: Box plots of quantified ginsenosides.

## Abbreviations

ANOVA	analysis of variance
ELSD	evaporative light-scattering detector
ESI	electrospray ionization
HPLC	high-performance liquid chromatography
HR-MAS	high-resolution magic angle spinning
HR-MAS	high-resolution magic angle spinning
LODs	limits of detection
MS	mass spectrometry
NMR	nuclear magnetic resonance
OPLS-DA	orthogonal partial least squares discriminant analysis
PCA	principal component analysis
PLS-DA	partial least squares discriminant analysis
PPD	protopanaxadiol
PPT	protopanaxatriol
QTOF	quadrupole time of flight
RT	retention time
