# Supplementary figures and images for: Comparative Analysis of Panax ginseng Berries from Seven Cultivars Using UPLC-QTOF/MS and NMR-Based Metabolic Profiling
Source: Biomolecules. 2019 Aug 28;9(9):424. doi: 10.3390/biom9090424 (PMC6770912; doi:10.3390/biom9090424)

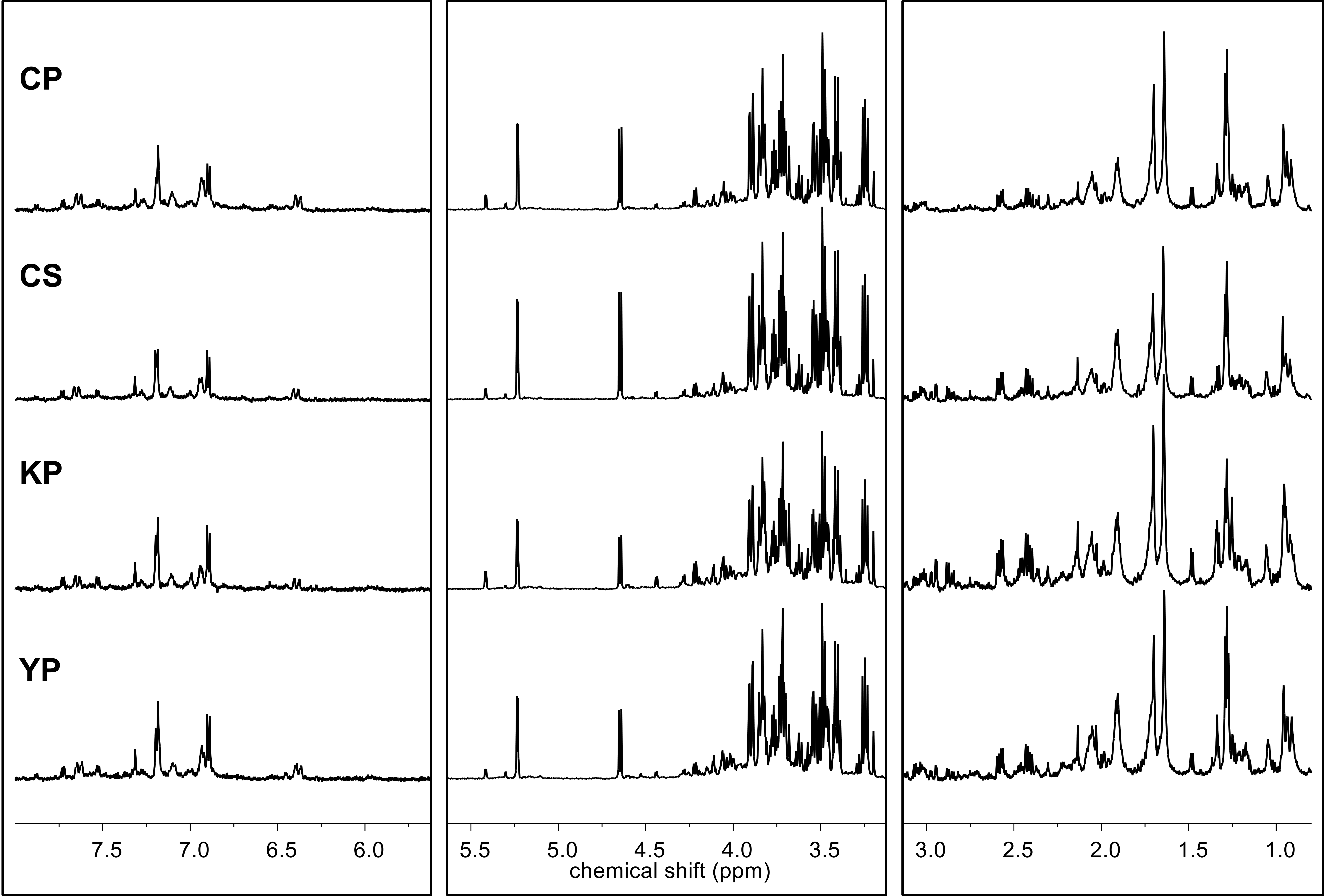

Supplement: Supplementary file 1 [file biomolecules-09-00424-s001.zip › Figure S1.tif]

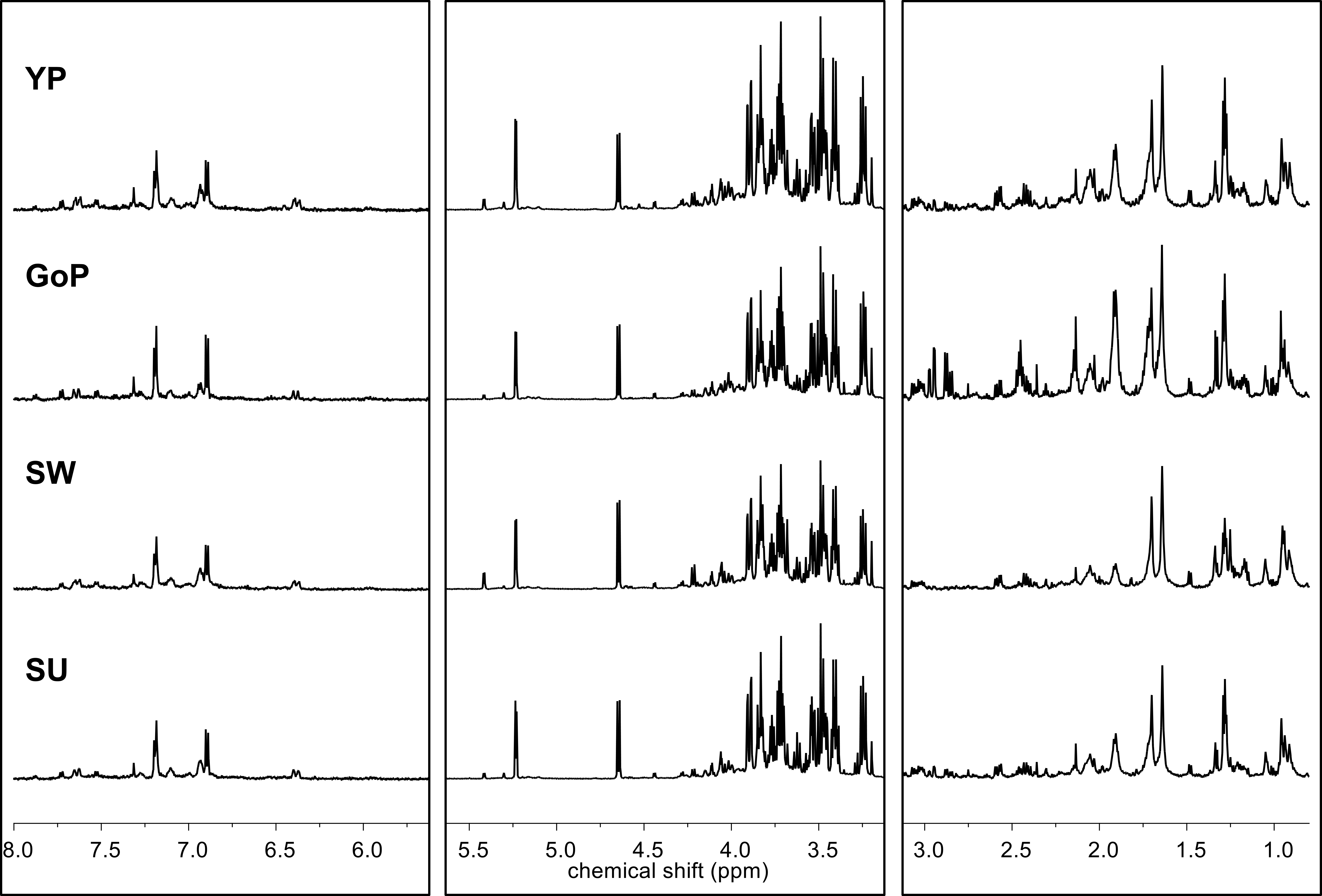

Supplement: Supplementary file 1 [file biomolecules-09-00424-s001.zip › Figure S2.tif]

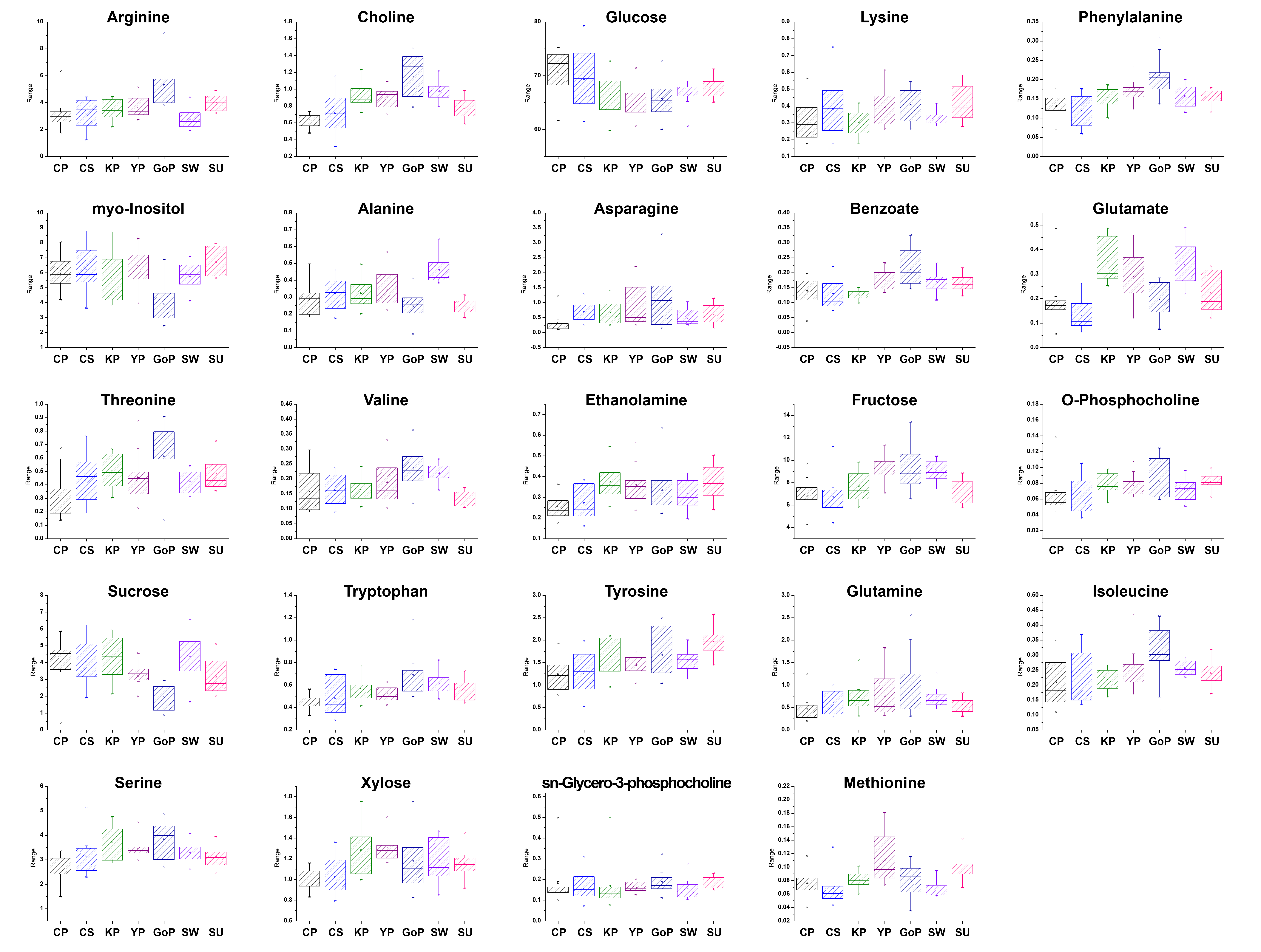

Supplement: Supplementary file 1 [file biomolecules-09-00424-s001.zip › Figure S3.tif]

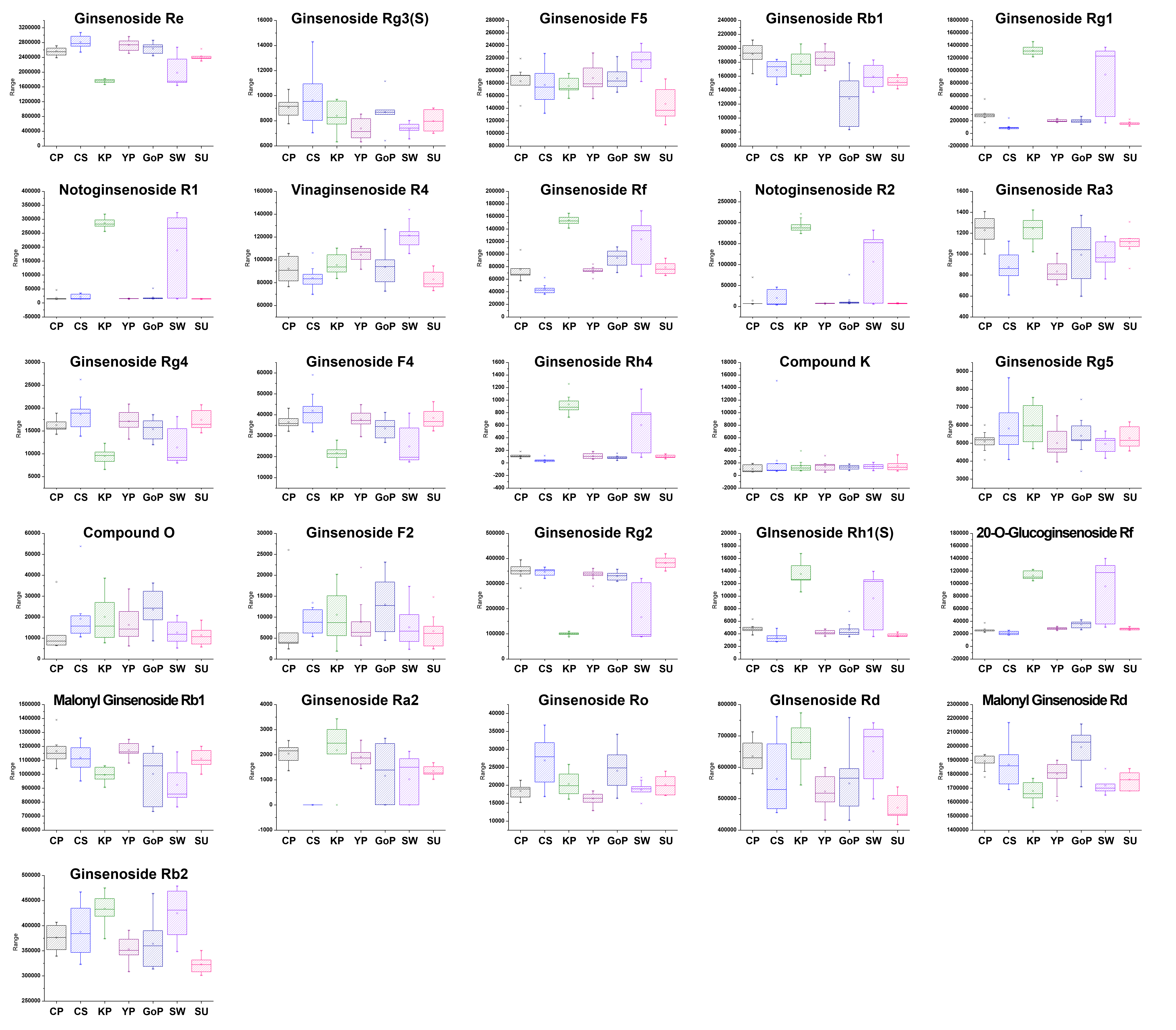

Supplement: Supplementary file 1 [file biomolecules-09-00424-s001.zip › Figure S4.tif]
